# Encoding Gaussian curvature in glassy and elastomeric liquid crystal solids

**DOI:** 10.1098/rspa.2016.0112

**Published:** 2016-05

**Authors:** Cyrus Mostajeran, Mark Warner, Taylor H. Ware, Timothy J. White

**Affiliations:** 1 Department of Engineering, University of Cambridge, Cambridge CB2 1PZ, UK; 2 Cavendish Laboratory, University of Cambridge, 19 JJ Thomson Avenue, Cambridge CB3 0HE, UK; 3 Materials and Manufacturing Directorate, Air Force Research Laboratory, Wright-Patterson Air Force Base, OH 45433, USA; 4 Department of Bioengineering, The University of Texas at Dallas, 800 W Campbell Road, Richardson, TX 75080, USA

**Keywords:** nematic, elastomers, solids, curvature

## Abstract

We describe shape transitions of thin, solid nematic sheets with smooth, preprogrammed, in-plane director fields patterned across the surface causing spatially inhomogeneous local deformations. A metric description of the local deformations is used to study the intrinsic geometry of the resulting surfaces upon exposure to stimuli such as light and heat. We highlight specific patterns that encode constant Gaussian curvature of prescribed sign and magnitude. We present the first experimental results for such programmed solids, and they qualitatively support theory for both positive and negative Gaussian curvature morphing from flat sheets on stimulation by light or heat. We review logarithmic spiral patterns that generate cone/anti-cone surfaces, and introduce spiral director fields that encode non-localized positive and negative Gaussian curvature on punctured discs, including spherical caps and spherical spindles. Conditions are derived where these cap-like, photomechanically responsive regions can be anchored in inert substrates by designing solutions that ensure compatibility with the geometric constraints imposed by the surrounding media. This integration of such materials is a precondition for their exploitation in new devices. Finally, we consider the radial extension of such director fields to larger sheets using nematic textures defined on annular domains.

## 1. Introduction

It is well known that inhomogeneous local deformations in thin solid sheets, for instance differential growth in leaves, can lead to the formation of Gaussian curvature and complex shape transitions [1]. We are concerned with modern responsive materials that can be preprogrammed to undergo prescribed spatially inhomogeneous expansions and contractions in response to external stimuli, offering exciting possibilities for the design and production of switchable surfaces for a variety of applications [2–6]. In particular, liquid crystal (LC) solids, either glassy or elastomeric, have orientational order about a director ***n***, but unlike their classical fluid analogues, they cannot flow. Nematic liquid crystalline glasses and elastomers are particularly promising candidates as responsive materials. They are richer than isotropic systems, for instance those with spatially varying capacity to swell, in that both the direction ***n*** of anisotropy as well as the degree of mechanical response are variable. Stimuli inducing such changes are typically light or heat, both of which can reduce the degree of order and cause contraction. The contraction λ<1 along ***n***, and elongation by λ^−*ν*^>1 in the directions perpendicular to ***n***, are large in elastomers, where the optothermal Poisson ratio [7] $\nu =\frac{1}{2}$ relates the perpendicular and parallel responses (with constancy of volume). Glasses have λ∼ 0.90–1.0 only, and their $\nu \in (\frac{1}{2},2)$ (volume increase).

Liquid crystal elastomers have a subtle mechanics, because director rotation can accompany some imposed deformation and there can be little or no stress—a remarkable response termed soft elasticity. Conceivably, soft elasticity could mask the Gaussian curvature we explore, except that (i) heating all the way to the isotropic phase eliminates order and hence anisotropy that can be redirected in order to relieve stress, and (ii) we find states that are unstretched with respect to the new metric, and there is little incentive for director rotation. We henceforth ignore soft elasticity and treat nematic glasses and elastomers in the same way, until we come to discuss actuation and device implications.

The spontaneous deformation gradient tensor in nematic glasses [8] and elastomers [9] is of the form

1.1 $$F=(\lambda -\lambda^{-\nu })n⊗n+\lambda^{-\nu }\;{\mathrm{Id}}_{3},$$

where Id_3_ denotes the identity operator on $R^{3}$. When *F* is achieved, the heated or irradiated body is in a relaxed state without stresses.

The distortions associated with heating a sheet with uniform ***n*** are quite spectacular, but more subtle and perhaps more useful effects are achieved with a programmed-in, non-uniform ***n***. Gaussian curvature arises, because the metric, specifying the intrinsic geometry of the deformed plane, spatially varies. The first example investigated [4] was that generated by concentric circles of ***n***. One immediately sees, if circumferences contract by a factor of λ<1 and radii extend by λ^−*ν*^>1, the circles now sit on what has morphed into a cone, concentric with its tip where the localized Gaussian curvature resides. Experimentally, effects are large, even in glasses [10], and still more so in elastomers [11]. These director circles are topological charge *q*=1 defects (disclinations) in a two-dimensional director field. Other charges instead give delocalized Gaussian curvature [7] of both signs. Programming ***n*** into LC sheets is possible at such precision that *q*=6 defects can be created in elastomers, and in the case of glasses, even *q*=±10 defects have been produced which generate highly complex surfaces [12,11]. In optics, such structures are used as axially symmetric wave plates [13], for instance with charge *q*=64, or termed ‘*q*-plates’, producing sophisticated light polarization, with charge *q*=100 being employed [14].

More general distributions of Gaussian curvature by variation of director in plane were attacked in the seminal work of Aharoni *et al.* [5] that describes the interplay between the nematic director field of a thin elastomeric sheet and the resulting three-dimensional configuration attained upon heating. In particular, they consider the difficult reverse problem of constructing a director field that induces a specified two-dimensional metric. In cases of one-dimensional variation (leading to surfaces of revolution), they have a direct way of finding the metric. These authors also go on to consider more ambitious problems such as two-dimensional variation. In this paper, we follow [6] and consider some specific in-plane director field patterns on thin nematic sheets that give interesting curvature distributions and consequent shapes that are then compared qualitatively with our experiments. We continue by considering spiral director patterns that produce more advanced shapes—cones, hyperbolic cones, (pseudo)spherical caps and spindles. An additional but important constraint is then added in—that the generated shape is geometrically compatible with a surrounding, inert sheet, so that the morphing structure can be anchored. Practical devices will require such attachment, as they respond to stimuli, in order to exploit induced Gaussian curvature to pump, do work or actuate. We will conclude by discussing how applications might be achieved because of this essential advance. We now review the mathematical framework [5,6] used in the subsequent analysis.

## 2. Generating intrinsically curved surfaces

### (a) Gaussian curvature through patterning

It is assumed that the director field does not vary across the thickness of the sheet, so that the same pattern is repeated at each level of thickness. For sufficiently thin sheets, stimulation of the system will result in pure bending of the sheet at no stretch energy cost and one expects an isometric immersion of the prescribed local deformations as determined by the director field pattern.

Let $(x_{1},x_{2})\in \omega \subset R^{2}$ be Cartesian coordinates parametrizing the midsurface of the initially flat sheet and $n(x_{1},x_{2})=n_{1}{\hat{e}}^{1}+n_{2}{\hat{e}}^{2}$ be the director field pattern across the surface, where ${\hat{e}}^{1}$, ${\hat{e}}^{2}$ form the standard orthonormal basis of $R^{2}$. The associated in-plane spontaneous deformation tensor *F* has components *F*_*αβ*_=(λ−λ^−*ν*^)*n*_*α*_*n*_*β*_+λ^−*ν*^*δ*_*αβ*_, where *α*, *β*=1,2. The resulting two-dimensional metric of the deformed sheet upon stimulation is *a*=*F*^T^*F*, which simplifies to

2.1 $$a_{\alpha \beta }=(\lambda^{2}-\lambda^{-2\nu })n_{\alpha }n_{\beta }+\lambda^{-2\nu }\delta_{\alpha \beta }.$$

The Gaussian curvature *K* of a surface is an intrinsic geometric property (*Theorema Egregium*) that is determined by the first fundamental form *a*_*αβ*_ of the surface via

2.2 $$K=-\frac{1}{a_{11}}(\partial_{1}\Gamma_{12}^{2}-\partial_{2}\Gamma_{11}^{2}+\Gamma_{12}^{1}\Gamma_{11}^{2}-\Gamma_{11}^{1}\Gamma_{12}^{2}+\Gamma_{12}^{2}\Gamma_{12}^{2}-\Gamma_{11}^{2}\Gamma_{22}^{2}),$$

where $\Gamma_{\mu \rho }^{\tau }=\frac{1}{2}a^{\tau \sigma }(\partial_{\mu }a_{\sigma \rho }+\partial_{\rho }a_{\mu \sigma }-\partial_{\sigma }a_{\mu \rho })$ and (*a*^*μρ*^)=(*a*_*μρ*_)^−1^.

Ignoring spatial variation in λ that would also contribute to the Christoffel symbols $\Gamma_{\mu \rho }^{\tau }$ and hence *K*,^1^ we concentrate on the two-dimensional director field that is characterized by an angle scalar field *ψ*=*ψ*(*x*_1_,*x*_2_) specifying the in-plane orientation of the director at each point on the initially flat sheet, so that $n_{1}=\cos\psi$ and $n_{2}=\sin\psi$. The Gaussian curvature determined by the nematic metric can be expressed in terms of *ψ* as

2.3 $$K=\frac{\lambda^{2\nu }-\lambda^{-2}}{2}[(\partial_{2}^{2}\psi -\partial_{1}^{2}\psi -4\partial_{1}\psi \partial_{2}\psi )\sin(2\psi )+2(\partial_{1}\partial_{2}\psi +{\left(\partial_{2}\psi \right)}^{2}-{\left(\partial_{1}\psi \right)}^{2})\cos(2\psi )].$$

We note here that if we rotate the director associated with a given pattern by *π*/2 in-plane at every point, so that ψ→ψ+π/2, then the resulting Gaussian curvature flips sign everywhere, because sin⁡2ψ→−sin⁡2ψ and cos⁡2ψ→−cos⁡2ψ. That is,

2.4 $$K\to -K\;\mathrm{as}\;\psi \to \psi +\frac{\pi }{2}.$$

We refer to a pair of director field patterns that are related by a *π*/2 radian rotation of the directors as orthogonal duals, pairs that relate to heating and cooling.^2^ In this paper we consider only λ≤1, that is heating; we achieve changes in sign of *K* by changing the *n*(*r*).

### (b) Spherical and pseudo-spherical surfaces

We now restrict our attention to director fields of the form $n=\cos\psi (x_{2})\;{\hat{e}}^{1}+\sin\psi (x_{2})\;{\hat{e}}^{2}$, whose alignment angle field varies only with respect to one of the coordinates. The Gaussian curvature upon stimulation is $K=-\frac{1}{2}(\lambda^{-2}-\lambda^{2\nu })(\psi^{\prime \prime }\sin2\psi +2\psi^{\prime 2}\cos2\psi ).$ We can rewrite this as

2.5 $$\frac{d^{2}}{dx_{2}^{2}}\cos2\psi =4\;C(K),$$

where *C*(*K*)=*K*/(λ^−2^−λ^2*ν*^), and solve for constant *K*>0 to find

2.6 $$\psi (x_{2})=\pm \frac{1}{2}\arccos(c_{1}+c_{2}x_{2}+2C(K)x_{2}^{2}),$$

where *c*_1_, *c*_2_ are constants of integration. This pattern generates constant Gaussian curvature *K* wherever it is well defined. Now, consider the particular solution

2.7 $$\psi (x_{2})=\pm \frac{1}{2}\arccos\left(2{\left(1-\frac{x_{2}}{L}\right)}^{2}-1\right),$$

corresponding to *c*_1_=1, *c*_2_=−4/*L*, and

2.8 $$K=\frac{\lambda^{-2}-\lambda^{2\nu }}{L^{2}}>0.$$

**Figure 1.**
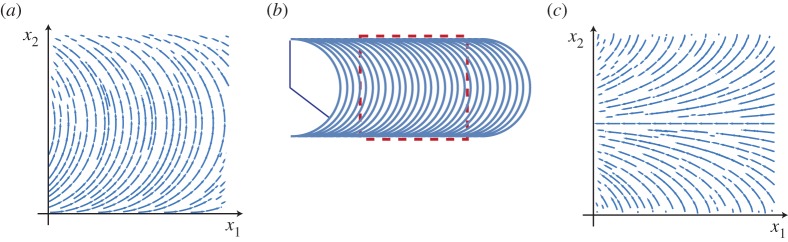
(*a*) The director field defined by $\psi (x_{2})=\arccos(1-x_{2}/L)$ on the square domain *ω*=[0,2*L*]×[0,2*L*]. This pattern generates constant Gaussian curvature *K*=(λ^−2^−λ^2*ν*^)/*L*^2^>0 upon stimulation. (*b*) The nematic pattern obtained by shifting a semicircular arc of radius $L=(1/\sqrt{K})\sqrt{\lambda^{-2}-\lambda^{2\nu }}$ along the *x*_1_-axis generates constant positive Gaussian curvature *K*>0 upon stimulation. (*c*) The director field defined by $\psi (x_{2})=\pi /2+\arccos$ (1−*x*_2_/*L*) on the square domain *ω*=[0,2*L*]×[0,2*L*]. This pattern generates constant negative Gaussian curvature *K*=−(λ^−2^−λ^2*ν*^)/*L*^2^<0. (Online version in colour.)

This can be rewritten as $\psi (x_{2})=\arccos(1-x_{2}/L),$ which describes a well-defined pattern for 0≤*x*_2_≤2*L*. The pattern on the square domain *ω*=[0,2*L*]×[0,2*L*] is shown in figure 1*a*. By integrating along the director field lines, we note that the integral curves of this pattern consist of semicircles of radius *L* that are shifted along the *x*_1_-axis. Thus, if we seek to encode a particular constant positive Gaussian curvature *K*=*K*_0_>0 across an initially flat sheet, then we can do so by encoding the pattern obtained by shifting a semicircle of radius $L=(1/\sqrt{K_{0}})\sqrt{\lambda^{-2}-\lambda^{2\nu }}$ as shown in figure 1*b*.

By the observation that the orthogonal dual of a given two-dimensional director field pattern generates the exact opposite Gaussian curvature at every point, we can encode constant negative Gaussian curvature *K*=−*K*_0_ on a thin nematic sheet by simply using the orthogonal dual of the pattern that encodes positive curvature *K*_0_>0. Returning to the example of figure 1*a*, where a pattern encoding constant positive curvature *K*=(λ^−2^−λ^2*ν*^)/*L*^2^ was defined on the square domain *ω*=[0,2*L*]×[0,2*L*] by $\psi (x_{2})=\arccos(1-x_{2}/L)$, we immediately obtain a pattern on the same domain which encodes constant negative Gaussian curvature *K*=−(λ^−2^−λ^2*ν*^)/*L*^2^, by simply taking $\psi (x_{2})=\arccos(1-x_{2}/L)+\pi /2$. The resulting pattern is shown in figure 1*c*. This pattern is generated by shifting a tractrix curve along its axis.

For a surface in $R^{3}$, the components *a*_*αβ*_ and *b*_*αβ*_ of the first and second fundamental forms satisfy a system of algebraic differential equations known as the Gauss–Codazzi–Mainardi equations. Conversely, any pair (*a*,*b*) consisting of a symmetric and positive definite matrix field (*a*_*αβ*_) and a symmetric matrix field (*b*_*αβ*_) that satisfy the Gauss–Codazzi–Mainardi equations determines a unique surface up to a rigid transformation in $R^{3}$ [15]. Thus, to determine the equilibrium configuration of the mid-surface of an initially flat nematic sheet upon stimulation, we also need to know the components *b*_*αβ*_ of the second fundamental form that minimize the bending energy subject to the Gauss–Codazzi–Mainardi constraints.

For a fixed two-dimensional metric, the problem of identifying equilibrium configurations that minimize the bending energy reduces to the problem of minimizing the *Willmore functional*

2.9 $$I_{W}=\int_{\omega }H^{2}\;dS,$$

where *H* is the mean curvature of the deformed surface, among isometric immersions of the given metric [16–18].

For a metric of constant positive Gaussian curvature, it is easy to show that the Willmore functional is minimized precisely for spherical solutions. That is, a flat nematic sheet whose director field encodes constant positive curvature *K* is expected to form part of a sphere of radius $R=1/\sqrt{K}$ upon stimulation, assuming that the sheet is small enough to exclude the possibility of self-intersection [6].

In the case of a metric of constant negative Gaussian curvature, identifying minimizers of the Willmore functional is considerably less straightforward. In [19], it is shown that for a hyperbolic elastic disc that has already undergone local deformations, surfaces that are geodesic discs lying on hyperboloids of revolution of constant Gaussian curvature are minimizers of the Willmore functional among *smooth* immersions of the metric. These solutions will appear as saddle shapes in experiments and are expected to be energetically favourable for sufficiently small discs. However, it has been shown numerically that certain non-smooth wavy surfaces formed as odd periodic extensions of subsets of so-called Amsler surfaces are energetically more favourable than the smooth saddle shapes that correspond to discs lying on hyperboloids of revolution when the radius of the hyperbolic disc is sufficiently large [20]. We synthesize LC solid films with spatially programmed directors in order to realize shape-changing surfaces of constant Gaussian curvature.

### (c) Experimental investigations

The director profile in the plane of nematic LC solid sheets can be programmed through a variety of methods, including mechanical and magnetic fields [21,22]. Using these methods, however, it is difficult to spatially control the director orientation. Here we use chemistry amenable to surface alignment: precursor molecules, to what will become the nematic solid sheet, have been specifically designed to align to treated surfaces. Using this approach, low molar mass nematic LC monomers are filled between two plates separated by a well-defined gap. The treated surfaces, on the interior of the plates, direct the ordering of the LCs along a specific orientation through the thickness of the material. By using reactive nematic mesogens, this director orientation can be trapped in an elastic solid. Spatially complex director patterns in LC cells are prepared using point-by-point photoalignment of an azobenzene dye by irradiation with polarized light [12,23]. By altering the polarization of the incident light, the in-plane orientation of the director of the LC can be spatially controlled. The resulting director field is a pixelated approximation of the desired smooth pattern with each pixel measuring 100×100 μm.

**Figure 2.**
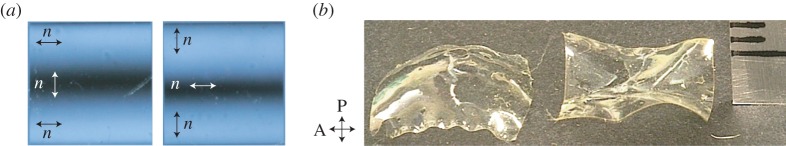
(*a*) Polarized optical images of the patterned director profiles predicted to generate positive (left) and negative (right) curvature. The patterns are optically equivalent between crossed polarizers. The director orientation at the edges and centre of the pattern is indicated with arrows. Each square film has a side length of 10 mm. (*b*) Positive (left) and negative (right) Gaussian curvature in 15 μm thick glassy LC solid film at 175°*C*. (Online version in colour.)

A number of glassy liquid crystalline solids have been demonstrated to be compatible with surface alignment techniques. Here we use one such composition with λ=0.94 and *ν*=0.92 [24]. Specifically, we use the composition with the lowest cross-link density from this work. This composition is representative of the larger class of nematic LC glasses that can be aligned using surface alignment techniques [25]. The director patterns depicted in figure 1*a*,*c* were chosen to assess the viability of generating Gaussian curvature on exposure to stimulus. After fabrication, the LC solid film is flat at 25°*C* and retains the expected birefringence of an aligned nematic, as seen in figure 2*a*.

The thermally induced shape change of the nematic LC glass is shown in figure 2*b*. As predicted, the pattern depicted in figure 1*a* leads to the formation of positive Gaussian curvature, whereas the pattern from figure 1*c* leads to negative Gaussian curvature. On removal of the heat, the film returns to a largely flat state. It should be noted that the positive Gaussian curvature sample exhibits a periodic buckling around a pair of oppositely faced edges of the film. This is likely owing to the relatively sharp change in director angle with respect to the resolution of the patterning technique near these edges of the film. This buckling highlights the limitation on the curvature that can be achieved in nematic LC glasses with comparatively small strains.

**Figure 3.**
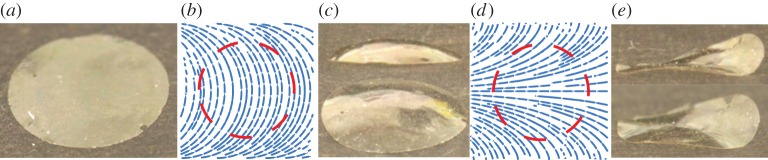
From left to right: (*a*) the initially flat configuration of a circular glassy film 15 μm in thickness and 7.1 mm in diameter. (*b*) The positive Gaussian curvature pattern. The dashed circle indicates the boundaries of the circular film. (*c*) The formation of positive Gaussian curvature in the actuated state from two distinct viewing angles. (*d*) The negative curvature pattern obtained as the orthogonal dual director field. (*e*) The formation of negative Gaussian curvature in the actuated state from two viewing angles. (Online version in colour.)

In order to improve the quality of the surfaces that are formed in stimulated nematic glasses, a discoid subsection of the patterned films was removed and exposed to stimulus, as shown in figure 3. In the case of films encoded with either of the identified patterns, the predicted smooth curvature is realized upon stimulation. Indeed, shape selection of the equilibrium surface in the positive curvature case seems to be in remarkable qualitative agreement with the predicted solution of a spherical cap. In the negative curvature case too, the equilibrium surface appears largely consistent with the hyperboloid saddle solution that is predicted to be energetically favourable for a glassy film at this scale. Owing to the tendency for buckling in areas of the film where the director changes rapidly with respect to the resolution of the patterning process, it should be noted that the curvature cannot be increased by simply scaling the pattern to smaller dimensions. Instead, higher strain materials are needed.

**Figure 4.**
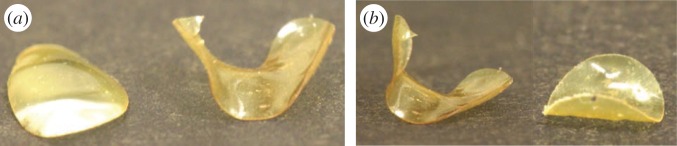
(*a*) Side-by-side comparison of positive (left) and negative (right) Gaussian curvature realization in actuated elastomeric films. (*b*) Comparison of shape selection of discs in the negative Gaussian curvature case depending on the size of the domain. For smaller radii (3.7 mm initial diameter), a saddle shape is formed as expected (right). When the radius of the disc is sufficiently large (7.1 mm initial diameter), considerably more complex surfaces with wavy edges are formed (left). (Online version in colour.)

To facilitate larger curvature realization, we prepare a comparatively high strain surface-alignable LC elastomer with λ=0.65 [23]. Using the pattern depicted in figure 1*a*, positive Gaussian curvature is encoded in the elastomeric film. As can be seen in figure 4*a*, the film encoded for positive Gaussian curvature forms part of a sphere with a slightly elliptical distortion. Figure 4*a* also shows a complexly buckled hyperbolic surface that is formed when an elastomeric disc encoded to give negative Gaussian curvature is exposed to stimulus. For smaller diameter films encoded with negative Gaussian curvature, a classic saddle shape can be observed, as shown in figure 4*b*. The surface that is formed by the larger radius hyperbolic disc of figure 4*b* might be interpreted as a distorted periodic Amsler surface. Comparatively, these deformations are significantly larger than those observed for the glassy films despite being more than three times as thick. Understanding the full spectrum of shape selection for films encoded with negative Gaussian curvature is an area of ongoing consideration.

These qualitative explorations can be made quantitative if (i) the λ values can be accurately known (results are very sensitive to λ), and (ii) accurate imaging from several vantage points is made, so that shell shapes and dimensions can be accurately fitted. This work, also requiring further synthesis, is underway.

## 3. Spiral director fields

### (a) Metric analysis in polar coordinates

Now, consider a director field $n=n_{1}{\hat{e}}^{1}+n_{2}{\hat{e}}^{2}$ whose components are given by

3.1 $$n_{1}(r,\theta )=\cos\Psi (r,\theta )\;\mathrm{and}\;n_{2}(r,\theta )=\sin\Psi (r,\theta ),$$

where (*r*,*θ*) are the polar coordinates on *ω*, and Ψ(r,θ)=ψ(rcos⁡θ,rsin⁡θ) is the alignment angle field expressed in (*r*,*θ*) coordinates. The metric components with respect to polar coordinates are

3.2 $$\left(\begin{array}{cc} a_{rr} & a_{r\theta }\\ a_{\theta r} & a_{\theta \theta } \end{array}\right)=J^{T}\left(\begin{array}{cc} a_{11} & a_{12}\\ a_{21} & a_{22} \end{array}\right)J,$$

where J is the Jacobian matrix

3.3 $$J=\left(\begin{array}{cc} \partial_{r}x_{1} & \partial_{\theta }x_{1}\\ \partial_{r}x_{2} & \partial_{\theta }x_{2} \end{array}\right).$$

In terms of the angle field *Ψ*, the metric components are

3.4 $$\begin{array}{rl} a_{rr} & =\lambda^{2}+(\lambda^{-2\nu }-\lambda^{2})\sin^{2}(\theta -\Psi ),\\ a_{r\theta } & =a_{\theta r}=\frac{r}{2}(\lambda^{-2\nu }-\lambda^{2})\sin2(\theta -\Psi )\\ \text{and}\;\;a_{\theta \theta } & =r^{2}[\lambda^{-2\nu }-(\lambda^{-2\nu }-\lambda^{2})\sin^{2}(\theta -\Psi )]. \end{array}\}$$

By Gauss's theorem, the Gaussian curvature is given by

3.5 $$K=-\frac{1}{a_{rr}}(\partial_{r}\Gamma_{r\theta }^{\theta }-\partial_{\theta }\Gamma_{rr}^{\theta }+\Gamma_{r\theta }^{r}\Gamma_{rr}^{\theta }-\Gamma_{rr}^{r}\Gamma_{r\theta }^{\theta }+\Gamma_{r\theta }^{\theta }\Gamma_{r\theta }^{\theta }-\Gamma_{rr}^{\theta }\Gamma_{\theta \theta }^{\theta }).$$

For nematic angle fields of the form *Ψ*(*r*,*θ*)=*θ*+*α*(*r*), the metric components in polar coordinates depend only on the radius *r*, and the Gaussian curvature is given by

3.6 $$K=\frac{\lambda^{-2}-\lambda^{2\nu }}{2}\left[\left(\alpha^{\prime \prime }+\frac{3}{r}\alpha^{\prime }\right)\sin(2\alpha )+2\alpha^{\prime 2}\cos(2\alpha )\right].$$

**Figure 5.**
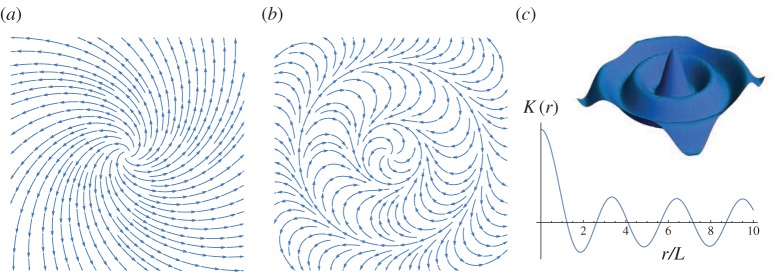
(*a*) A logarithmic spiral nematic pattern with *α*=*π*/4. (*b*) The director field defined by *α*(*r*)=*r*/*L* and (*c*) the resulting Gaussian curvature distribution *K*=*K*(*r*). (Online version in colour.)

The director field takes the form $n=\cos(\theta +\alpha )\;{\hat{e}}^{1}+\sin(\theta +\alpha )\;{\hat{e}}^{2}=\cos\alpha (r)\;{\hat{e}}_{r}+\sin\alpha (r)\;{\hat{e}}_{\theta },$ where ${\hat{e}}_{r}$ and ${\hat{e}}_{\theta }$ are the unit radial and azimuthal vectors, respectively. Note that *α* is the angle that the director makes with the radial direction. For logarithmic spiral patterns, this angle is constant, so that, by (3.6), the Gaussian curvature generated by a director field whose integral curves consist of logarithmic spirals vanishes everywhere except at the point defect at the origin. Figure 5*a* shows a logarithmic spiral pattern with *α*=*π*/4.

By prescribing *α*=*α*(*r*), we can obtain director fields that generate radially symmetric Gaussian curvature distributions *K*=*K*(*r*). An example with *α*(*r*)=*r*/*L* is shown in figure 5*b*. The Gaussian curvature, in this case, is given by

3.7 $$K(r)=\frac{\lambda^{-2}-\lambda^{2\nu }}{2}\left[\frac{2}{L^{2}}\cos\left(\frac{2r}{L}\right)+\frac{3}{Lr}\sin\left(\frac{2r}{L}\right)\right].$$

For constant K∈R, (3.6) can be viewed as an ODE in *α* which is solved by

3.8 $$\alpha (r)=\pm \frac{1}{2}\arccos\left(-C(K)\frac{r^{2}}{2}+c_{1}+\frac{c_{2}}{r^{2}}\right),$$

where *C*(*K*)=*K*/(λ^−2^−λ^2*ν*^), and *c*_1_, *c*_2_ are real constants of integration. Depending on the choice of *c*_1_ and *c*_2_, these solutions define curvature-inducing nematic patterns on compact discs or annular domains. If *K*>0, *c*_2_=0 and *c*_1_≤1, then the solution (3.8) defines a spiral pattern on the compact disc

3.9 $$r\leq \sqrt{\frac{2(1+c_{1})}{C(K)}}.$$

**Figure 6.**
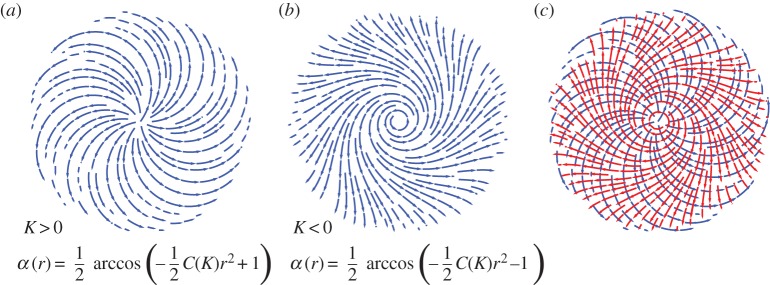
Constant-curvature-inducing nematic spiral patterns on discs of maximal radius $r_{D}=2/\sqrt{|C(K)|}$. (*a*) The director field of the nematic spiral pattern which generates prescribed constant curvature *K*>0. (*b*) The director field of the nematic spiral pattern which generates prescribed constant curvature *K*<0. (*c*) Orthogonal duality of constant-curvature-inducing spirals of opposite sign on a disc of maximal radius. The spiral pattern (red) whose integral curves turn in a clockwise direction away from the origin generates negative Gaussian curvature. (Online version in colour.)

Note that on the boundary of this disc, the expression for *α* in (3.8) attains its maximum value of *α*=*π*/2. For *c*_1_=1, we obtain a spiral pattern on a disc of maximal radius, which encodes constant positive Gaussian curvature *K*. This gives the largest disc on which a smooth radially symmetric director field can be used to encode a prescribed constant Gaussian curvature. Attempting to increase the radius beyond this point by varying *c*_1_ as a parameter leads to a bifurcation at which the solution domain becomes annular instead of discoid. If *K*<0, then it is *c*_2_=0 and *c*_1_=−1 which yield the spiral pattern of maximal radius. These patterns are shown in figure 6*a*,*b*.

In the light of the result that the orthogonal dual of a director field has precisely the opposite Gaussian curvature distribution, we find that the spirals in figure 6 make perfect sense. In particular, the spiral in figure 6*b* is equivalent to the spiral that turns in the opposite direction, defined by an $\alpha (r)=-\frac{1}{2}\arccos(-C(K)r^{2}/2-1)$. This equivalent pattern is the orthogonal dual of the positive curvature spiral of figure 6*a* and is illustrated in figure 6*c*, noting *C*(*K*) has reversed sign.

Finally, note that for *K*=0 and the choice of *c*_2_=0, solution (3.8) reduces to a logarithmic spiral pattern defined by *α*=constant. It should be noted, however, that the resulting surfaces upon stimulation will typically not remain planar. In particular, such patterns are expected to form cone and anti-cone equilibrium configurations upon stimulation [4,7,26,27].

### (b) Logarithmic spiral patterns: cone/anti-cone formation

It is shown in [7,4] that azimuthal (*α*=*π*/2) and radial (*α*=0) director fields generate cone and anti-cone surfaces upon stimulation, respectively. It is also argued there that logarithmic spiral patterns result in cone and anti-cone surfaces depending on whether the angle *α* is greater than or less than some threshold angle depending on λ and *ν*. Here, we will rederive this result using the nematic metric in polar coordinates. Generally, the angles between curves on a patterned nematic sheet become distorted upon stimulation. In particular, the radial curves on the initial sheet will not in general be orthogonal to the images of the azimuthal curves upon stimulation. We note that two curves *Γ*_1_ and *Γ*_2_ with polar parametric representations (*r*,*θ*)=(*r*_1_(*t*),*θ*_1_(*t*)) and (*r*,*θ*)=(*r*_2_(*t*),*θ*_2_(*t*)) are orthogonal with respect to the metric d*s*^2^=*a*_*rr*_ d*r*^2^+2*a*_*rθ*_ d*r* d*θ*+*a*_*θθ*_ d*θ*^2^, if and only if

3.10 $$a_{rr}r_{1}^{\prime }r_{2}^{\prime }+a_{r\theta }r_{1}^{\prime }\theta_{2}^{\prime }+a_{\theta r}\theta_{1}^{\prime }r_{2}^{\prime }+a_{\theta \theta }\theta_{1}^{\prime }\theta_{2}^{\prime }=0.$$

The concentric circles on the undeformed sheet can be parametrized such that *r*_1_′=0 and *θ*_1_′=1. Thus, the curve (*r*(*t*),*θ*(*t*)) on the undeformed sheet whose image becomes orthogonal to the images of the concentric circles upon stimulation satisfies *a*_*θr*_*r*′+*a*_*θθ*_*θ*′=0. That is, d*θ*/d*r*=−*a*_*θr*_/*a*_*θθ*_. The length of such a curve emanating from the origin is given by

3.11 $$l_{1}=\int_{0}^{r}\sqrt{a_{rr}+2\left(\frac{d\theta }{dr}\right)a_{r\theta }+{\left(\frac{d\theta }{dr}\right)}^{2}a_{\theta \theta }}\;dr$$

upon stimulation. Because d*θ*/d*r*=−*a*_*θr*_/*a*_*θθ*_, (3.11) reduces to

3.12 $$l_{1}=\int_{0}^{r}\frac{\sqrt{a_{rr}a_{\theta \theta }-a_{r\theta }^{2}}}{\sqrt{a_{\theta \theta }}}\;dr=\lambda^{1-\nu }\int_{0}^{r}\frac{r\;dr}{\sqrt{a_{\theta \theta }}}.$$

The perimeter of the closed curve that the circle of radius *r* deforms into following exposure to stimulus is given by

3.13 $$l_{2}=\int_{0}^{2\pi }\sqrt{a_{\theta \theta }(r)}\;d\theta =2\pi \sqrt{a_{\theta \theta }(r)}.$$

The analysis so far is valid for all angle fields of the form *Ψ*=*θ*+*α*(*r*), where $a_{\theta \theta }=r^{2}[\lambda^{-2\nu }-(\lambda^{-2\nu }-\lambda^{2})\sin^{2}\alpha ]$ by (3.4).

For logarithmic spirals, a cone is expected to form if 2*πl*_1_>*l*_2_ and an anti-cone is expected to form if 2*πl*_1_<*l*_2_. When a cone is formed, the cone angle is

3.14 $$\phi =\arcsin[\lambda^{-(1+\nu )}-(\lambda^{-(1+\nu )}-\lambda^{1+\nu })\sin^{2}\alpha ].$$

For 2*πl*_1_=*l*_2_, the sheet is expected to remain flat (*ϕ*=*π*/2) upon stimulation and the threshold angle *α*_*c*_ at which this holds satisfies $\lambda^{-2\nu }-(\lambda^{-2\nu }-\lambda^{2})\sin^{2}\alpha_{c}=\lambda^{1-\nu }$. That is,

3.15 $$\alpha_{c}=\arcsin\left(\sqrt{\frac{\lambda^{-2\nu }-\lambda^{1-\nu }}{\lambda^{-2\nu }-\lambda^{2}}}\right),$$

which is in agreement with [4]. We also note that we can choose the angle *α* of a logarithmic director field such that the lengths of azimuthal curves remain fixed. This is achieved by requiring $\sqrt{a_{\theta \theta }(r)}=r$, which is satisfied from (3.4) when

3.16 $$\alpha_{0}=\arcsin\left(\sqrt{\frac{\lambda^{-2\nu }-1}{\lambda^{-2\nu }-\lambda^{2}}}\right).$$

If *α*_*c*_<*α*_0_, then a cone is formed and as a result the symmetry of revolution is unbroken. This condition is equivalent to λ^1−*ν*^>1; i.e. *ν*>1. In particular, a logarithmic spiral director field with *α*=*α*_0_ patterned across the surface of a disc will keep the circular boundary fixed, whereas the interior of the surface rises to form a cone provided *ν*>1. The cone angle in this case is $\phi =\arcsin(\lambda^{\nu -1})$.

### (c) Spherical spindles

Here we extend the analysis presented in §3b to study the formation of spherical caps and spherical spindles using the positive-curvature-inducing spiral patterns defined by

3.17 $$\alpha (r)=\frac{1}{2}\arccos(-\frac{1}{2}C(K)r^{2}+c),$$

for a constant *c*. Spherical spindles [28,29] are surfaces of revolution of constant positive Gaussian curvature *K*. The top half of a spherical spindle can be parametrized as

3.18 $$r(s,t)=(\gamma_{1}(s)\cos t,\gamma_{1}(s)\sin t,\gamma_{2}(s)),$$

where the profile curve ***γ***(*s*)=(*γ*_1_(*s*),*γ*_2_(*s*)) is defined by

3.19 $$\gamma_{1}(s)=\rho \sin(s\sqrt{K})$$

and

**Figure 7.**
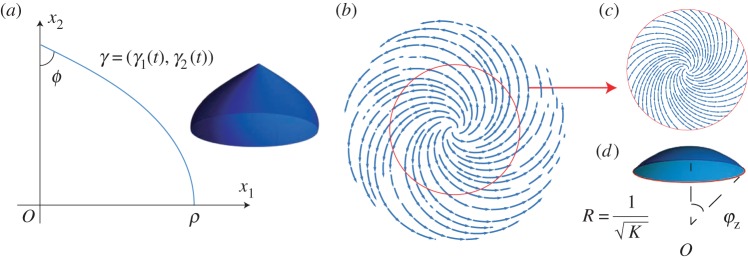
(*a*) The top half of a spindle of constant Gaussian curvature *K*>0 and parameter $\rho \in (0,1/\sqrt{K})$. The spindle arises as the surface of revolution obtained by revolving the profile curve *γ* about the *x*_2_-axis. (*b*) The director field defined by (3.17) with *K*>0 and *c*=1−2/(1+λ^1+*ν*^). The solid red curve indicates the circle of radius *r*_0_ whose length is unchanged by the pattern. (*c*) The director field on a disc of radius *r*_0_ and (*d*) the spherical cap of fixed boundary that is expected to form. (Online version in colour.)

3.20 $$\gamma_{2}(s)=\int_{0}^{\pi /2-s\sqrt{K}}\sqrt{\frac{1}{K}-\rho^{2}\sin^{2}\tau }\;d\tau ,$$

for $0<\rho \leq 1/\sqrt{K}$ and $s\in [0,\pi /2\sqrt{K}]$. Figure 7*a* illustrates one such surface and its profile curve. Note that for $\rho =1/\sqrt{K}$, the spindle reduces to a sphere of radius $1/\sqrt{K}$.

The length of each curve from the apex that intersects the azimuthal circles orthogonally is

3.21 $$l_{1}(s)=\int_{0}^{s}\sqrt{\gamma_{1}^{\prime 2}+\gamma_{2}^{\prime 2}}\;d\tau =s,$$

because *γ*_1_′^2^+*γ*_2_′^2^=1. Using (3.12) we also have

3.22 $$l_{1}=s(r)=\int_{0}^{r}\frac{\lambda^{1-\nu }r\;dr}{\sqrt{a_{\theta \theta }(r)}}.$$

By (3.17), we find that *a*_*θθ*_=*r*^2^(*μ*_1_−*μ*_2_*r*^2^), where

3.23 $$\mu_{1}=\frac{1}{2}[\lambda^{-2\nu }+\lambda^{2}+c(\lambda^{-2\nu }-\lambda^{2})]\;\mathrm{and}\;\;\mu_{2}=\frac{1}{4}\lambda^{2-2\nu }K.$$

Hence, (3.22) can be rearranged and exploited to get *r*(*s*):

3.24 $$\begin{array}{rl} s(r) & =\int_{0}^{r}\frac{\lambda^{1-\nu }\;dr}{\sqrt{\mu_{1}-\mu_{2}r^{2}}}\\ & =\frac{\lambda^{1-\nu }}{\sqrt{\mu_{2}}}\arctan\left(\frac{\sqrt{\mu_{2}}\;r}{\sqrt{\mu_{1}-\mu_{2}r^{2}}}\right), \end{array}$$

so that $r(s)=\sqrt{\mu_{1}/\mu_{2}}\sin(\frac{1}{2}\sqrt{K}s)$. The length of the image of the circle of radius *r* centred at the origin in the undeformed sheet becomes $l_{2}=2\pi r\sqrt{\mu_{1}-\mu_{2}r^{2}}$ upon stimulation. Thus, we find that

3.25 $$\frac{1}{2\pi }l_{2}(s)=r(s)\sqrt{\mu_{1}-\mu_{2}\;r{\left(s\right)}^{2}}=\frac{\mu_{1}}{\lambda^{1-\nu }\sqrt{K}}\sin s\sqrt{K}.$$

By comparing the circumference *l*_2_ with 2*πγ*_1_, the spindle radius being (3.19), we identify $\lambda^{\nu -1}\mu_{1}/\sqrt{K}$ with *ρ*. Given $\rho \leq 1/\sqrt{K}$, the deformed sheet is consistent with a spherical spindle precisely when λ^*ν*−1^*μ*_1_<1. That is, a spindle is expected to form if

3.26 $$c<\frac{2\lambda^{1+\nu }-\lambda^{2(1+\nu )}-1}{1-\lambda^{2(1+\nu )}}=1-\frac{2}{1+\lambda^{1+\nu }}.$$

The angle of the spindle at the apex is $\phi =\arcsin(\gamma_{1}^{\prime }(0))=\arcsin(\mu_{1}/\lambda^{1-\nu })$. That is,

3.27 $$\phi =\arcsin\left[\frac{1+c-(c-1)\lambda^{2+2\nu }}{2\lambda^{1+\nu }}\right].$$

If *c*=1−2/(1+λ^1+*ν*^), then a spherical cap of radius $1/\sqrt{K}$ will form instead.

For any given choice of the parameter *c*, the spiral in figure 6*a* keeps the circumference of an azimuthal circle of radius *r*=*r*_0_ fixed provided that it satisfies $\sqrt{a_{\theta \theta }(r)}=r$; see (3.13). That is, *r*_0_ satisfies $\sqrt{\mu_{1}-\mu_{2}r_{0}^{2}}=1,$ so that

3.28 $$r_{0}=\sqrt{\frac{\mu_{1}-1}{\mu_{2}}}=\frac{\sqrt{2}}{\lambda \sqrt{K}}\sqrt{1+c-\lambda^{2\nu }(2+(c-1)\lambda^{2})}.$$

In the case, *c*=1−2/(1+λ^1+*ν*^), corresponding to a spherical cap, we obtain

3.29 $$r_{0}=\frac{2}{\sqrt{K}}\sqrt{\lambda^{\nu -1}-\lambda^{2(\nu -1)}}.$$

Note that we require *ν*>1 for *r*_0_ to be well-defined and positive. We have thus identified a director field on a thin disc that will cause it to form a spherical cap with the same fixed circular boundary upon exposure to stimulus, as shown in figure 7*b*–*d*. The zenith angle *φ*_*z*_ of the resulting spherical cap is

3.30 $$\varphi_{z}=\sqrt{K}s(r_{0})=2\arctan\sqrt{\lambda^{1-\nu }-1}.$$

Note that even for strains as small as λ=0.98 and an optothermal Poisson ratio of *ν*=2, the zenith angle of the resulting spherical cap can be as large as approximately 16°. So we see that this method allows for rather prominent spherical caps to form even in the presence of the small strains observed in nematic glasses. The same methods can be used to specify patterns that generate spindles with unchanged circular boundaries.

### (d) Hyperbolic cones

We can perform a similar analysis in the case of the negative-curvature-inducing spiral patterns. The family of surfaces that form the negative curvature analogue of spherical spindles are hyperbolic cones [28], which are cone-like surfaces of revolution of constant negative Gaussian curvature away from the non-smooth tip of the cone. A hyperbolic cone of constant negative Gaussian curvature *K*<0 admits a parametrization

3.31 $$r(s,t)=(\gamma_{1}(s)\cos t,\gamma_{1}(s)\sin t,\gamma_{2}(s)),$$

where the profile curve ***γ***(*s*)=(*γ*_1_(*s*),*γ*_2_(*s*)) is defined by

3.32 $$\gamma_{1}(s)=\rho \sinh(s\sqrt{|K|})$$

and

3.33 $$\gamma_{2}(s)=\int_{0}^{s\sqrt{|K|}}\sqrt{\frac{1}{|K|}-\rho^{2}\cosh^{2}\tau }\;d\tau$$

for $0<\rho \leq 1/\sqrt{|K|}$ and

3.34 $$0\leq s\leq \frac{1}{\sqrt{|K|}}\mathrm{arsinh}\frac{\sqrt{1/|K|-\rho^{2}}}{\rho }.$$

Note that *γ*_2_ can also be expressed in terms of the incomplete elliptic integral of the second kind *E*, as

3.35 $$\gamma_{2}(s)=-i\sqrt{\frac{1}{|K|}-\rho^{2}}E\left(is\sqrt{|K|}|\;\frac{-\rho^{2}}{1/|K|-\rho^{2}}\right),$$

where

3.36 $$E(\beta \;|\;k^{2})=\int_{0}^{\beta }\sqrt{1-k^{2}\sin^{2}\tau }\;d\tau .$$

**Figure 8.**
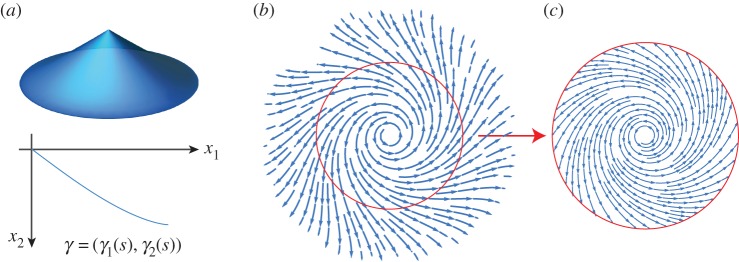
(*a*) A hyperbolic cone and its profile curve *γ*. (*b*,*c*) The director field defined by (3.17) with *K*<0 and *c*=−1. The solid (red) circle indicates the circle of radius *r*_0_ whose length is unchanged by the stimulation of the pattern. (Online version in colour.)

Figure 8*a* illustrates a typical hyperbolic cone and its profile curve arising from a negative curvature spiral.

Using the spiral pattern specified by (3.17) with prescribed constant *K*<0, the analysis proceeds as in the case of positive Gaussian curvature. The expression in (3.24) is modified to

3.37 $$\begin{array}{rl} s(r) & =\int_{0}^{r}\frac{\lambda^{1-\nu }\;dr}{\sqrt{\mu_{1}+|\mu_{2}|r^{2}}}\\ & =\frac{\lambda^{1-\nu }}{\sqrt{|\mu_{2}|}}\mathrm{arsinh}\left(r\sqrt{\frac{|\mu_{2}|}{\mu_{1}}}\right), \end{array}$$

where *μ*_1_ and *μ*_2_ are as in (3.23) with *K*<0. This yields $r(s)=\sqrt{\mu_{1}/|\mu_{2}|}\sinh(\frac{1}{2}\sqrt{|K|}s)$, and thus

3.38 $$\frac{1}{2\pi }l_{2}(s)=r\sqrt{\mu_{1}+|\mu_{2}|r^{2}}=\frac{\mu_{1}}{\lambda^{1-\nu }\sqrt{|K|}}\sinh s\sqrt{|K|}.$$

We see that this coincides with (3.32), using *γ*_1_ as a spindle radius to generate a circumference, if we identify $\lambda^{\nu -1}\mu_{1}/\sqrt{|K|}$ with *ρ*, whereby we note that the deformed sheet is consistent with a hyperbolic cone precisely if λ^*ν*−1^*μ*_2_<1; i.e. if *c*<1−2/(1+λ^1+*ν*^).

For any given choice of the parameter *c*, the spiral in figure 6*b* keeps the circumference of an azimuthal circle of radius *r*=*r*_0_ fixed provided that it satisfies $\sqrt{a_{\theta \theta }(r)}=r$. That is, *r*_0_ must satisfy $\sqrt{\mu_{1}+|\mu_{2}|r_{0}^{2}}=1$, so that

3.39 $$r_{0}=\sqrt{\frac{1-\mu_{1}}{|\mu_{2}|}}=\frac{\sqrt{2}}{\lambda \sqrt{|K|}}\sqrt{\lambda^{2\nu }(2+(c-1)\lambda^{2})-(1+c)}.$$

Note that *r*_0_ must also satisfy $r_{0}\leq r_{\max}(c)$, where

3.40 $$r_{\max}(c)=\frac{1}{\sqrt{|K|}}\sqrt{2(1-c)(\lambda^{-2}-\lambda^{2\nu })}$$

is the maximum radius on which (3.17) is well defined for a prescribed *K*<0. Figure 8*b* shows the negative curvature spiral pattern defined by (3.17) with *c*=−1. The solid (red) circle superimposed on the director field indicates the circle of radius *r*_0_ whose length is unchanged. The pattern in figure 8*c* is thus expected to generate a hyperbolic cone of fixed boundary upon stimulation.

### (e) Annular extensions of curvature-inducing spiral patterns

**Figure 9.**
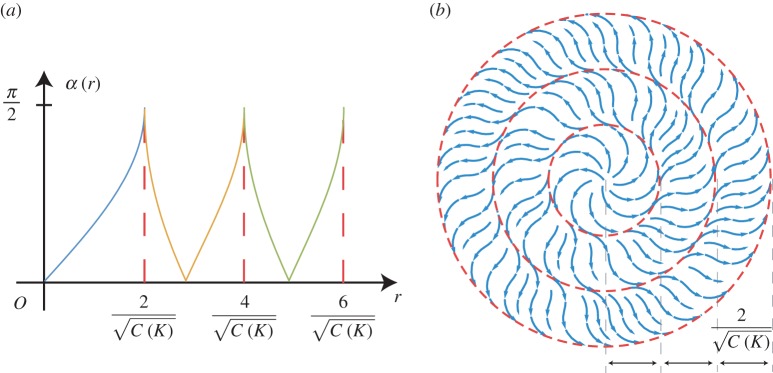
Extension of the positive curvature encoding spiral pattern using solutions in the form of (3.8) defined on annular domains. (*a*) The angle *α* that the directors make with the radial direction plotted against the radius *r*. An extension using two annular solutions is shown. (*b*) A visualization of the corresponding nematic director field. (Online version in colour.)

Finally, we note that it is possible to radially extend the constant curvature encoding spiral patterns of figure 6 to cover larger domains by the use of director fields defined on an annulus whose inner radius matches that of the disc. We can do so consistently through judicious choices of *c*_1_ and *c*_2_≠0 in the solution (3.8) for *α*, to ensure that the directors agree on the circular boundary between the disc and the first annulus. We can then repeat the process and extend the resulting pattern further by attaching another solution (3.8) defined on a second annulus whose inner radius matches the outer radius of the first annulus using a suitable new choice of *c*_1_ and *c*_2_. This process can be continued to produce director fields that encode constant Gaussian curvature on circular domains of any desired size. The resulting Gaussian curvature is well defined at all points on the domain with the exception of the boundaries where the different textures meet. Figure 9 depicts the nematic director field of the extended pattern and shows how the angle function *α*=*α*(*r*) varies under such an extension scheme for the *K*>0 spiral. The orthogonal dual of the extended pattern provides the corresponding result in the case of negative Gaussian curvature.

## 4. Discussion

In general, surfaces morphing to Gaussian-curved forms offer rich possibilities, most especially for delivering work as microdevices. Blocking the stroke of such morphing surfaces creates stretch rather than bend. Normally, extensile spontaneous strains of thin structures, when blocked lead to Euler instabilities and potentially strong processes become weaker bend actions. A Gaussian-curved structure is less likely to find such soft alternative routes. Impeding motion of the tip of an emerging cone, and thus resisting the contraction of circumferences lead to large associated forces, and these thus consequently do work, or perhaps pump an enclosed fluid. One might term this action ‘strong actuation’ as opposed to a weak actuation associated with bend. Another advantage is that which is often a short stroke (associated e.g. with a 4% length change in a glass) is larger in a morphing shell, for instance in the rise of a cone.

To avoid the nugatory effects of soft elasticity relieving strong tensile forces during actuation by director rotation, one must only have such stresses along the director. Often this can be achieved, for instance when a cone rising on heating is blocked, the circumferential stresses arising cannot be negated by director rotation, because stresses are already aligned along the director. However, more complex director distributions may be less reliable and it would be safer to use elastomers heated to isotropy, or glasses where there is no director rotation.

The anchoring of deforming shells to unresponsive mounts surrounding them is of vital importance for exploiting shells with tuneable Gaussian curvature. We have given explicit forms for the director distribution to achieve deformations, of both signs of curvature, elastically compatible with rigid connections to a mounting at the shell boundary. Negative curvature surfaces might be of practical advantage because they have sharp tips and are thus more acutely sensed, for instance, in haptic devices. Further, the slopes of their profile curves decrease with increasing radius, in contrast to cones. Thus, at their fixed boundaries where they could be mounted, there is less of a difference in angle and thus lower elastic energy in the attachment region. It is, however, impossible to design the pattern so as to ensure that the radius at which the boundary is fixed precisely matches the radius at which the profile curve fully flattens.

In summary, our results clearly indicate that Gaussian curvature can be realized in both low-strain, high-modulus glassy and high-strain, low-modulus elastomeric LC solid surfaces using appropriate, smooth, in-plane director fields patterned across initially flat films. Our preliminary experimental investigations show that patterned nematic solids may indeed be promising candidates for applications. In particular, the observed shape transformations were in qualitative agreement with theoretical predictions and the behaviour of the films in response to stimulus was found to be robust and reproducible. We hope that our results will encourage and stimulate further experimental research in achieving desired shape transitions in nematic LC solid surfaces. In particular, our discovery of anchorable structures should lead to further work in assessing the viability of morphing shells for specific applications.

## Footnotes

1 Indeed, λ(*r*) variation, but in isotropic systems without a director, is the basis of Gaussian curvature by spatially dependent swelling [2,3].

2 Heating the orthogonal dual to give a λ<1 along its director corresponds to cooling the original pattern to give an elongation along its director of *Λ*=λ^−*ν*^>1. Thus, heating and cooling give converse Gaussian curvatures.

## Data accessibility

All experimental data in this work (photographs, experimental conditions, etc.) are given in the text. All theoretical/computational data are also given in the text and relate to plots of mathematical results derived analytically.
